# The DRD2/ANKK1 Taq1A polymorphism in CYP2D6 extensive metabolizers is associated with the severity of extrapyramidal side effects of haloperidol treatment in schizophrenia spectrum disorders patients

**DOI:** 10.1192/j.eurpsy.2021.346

**Published:** 2021-08-13

**Authors:** A. Kibitov, E. Kiryanova, L. Salnikova, E. Zmeyeva, A. Shmukler, A. Kibitov

**Affiliations:** 1 Translational Psychiatry Department, V.M. Bekhterev National Medical Research Center for Psychiatry and Neurology, Saint-Petersburg, Russian Federation; 2 Moscow Research Institute For Psychiatry, Serbsky National Medical Research Center on Psychiatry and Addictions, Moscow, Russian Federation; 3 Laboratory Of Molecular Genetics, Serbsky National Medical Research Center on Psychiatry and Addictions, Moscow, Russian Federation

**Keywords:** Antipsychotics, Haloperidol, Pharmacogenetics

## Abstract

**Introduction:**

Schizophrenia is one of the most severe mental disorders. Haloperidol and other first-generation antipsychotics are widely used for schizophrenia treatment, but have prominent side effects, primarily extrapyramidal symptoms (EPS). The EPS severity is highly variable and may be underlied by genetic factors.

**Objectives:**

We performed a prospective study to test the association of DRD2/ANKK1 Taq1A polymorphism (rs18000497) and CYP2D6 phenotype, predicted from genotypes using 8 CYP2D6 alleles (*3, *4, *5, *6,*9, *10,*41, xN) with EPS severity during haloperidol treatment in schizophrenia spectrum disorders patients.

**Methods:**

57 inpatients with schizophrenia spectrum disorders (42,1% females; mean age - 46,7±11,8 y.o (M±SD) of European ancestry were enrolled in the study. Abnormal Involuntary Movement Scale (AIMS), Barnes Akathisia Rating Scale (BARS), Simpson-Angus Scale (SAS) were used to assess EPS on two timepoints: day 1 and day 21 of haloperidol treatment.

**Results:**

TaqIA T-allele carriers in contrast to wild-type allele homozygous patients had higher scores of BARS (p=0.029) and SAS (p=0.024) on day 21. After stratification by CYP2D6 phenotype, these differences were observed only in extensive metabolizers (p=0.006 and p=0.001 respectively), although the CYP2D6 phenotype itself was not associated with EPS severity. The combined effect of TaqIA T allele with CYP2D6 extensive phenotype on BARS score on day 21 was confirmed by General Linear Model (p=0.013).

**Conclusions:**

Our results show that minor TaqIA T-allele is associated with the severity of EPS after 3 weeks of haloperidol treatment only in CYP2D6 extensive metabolizers. That highlights the importance of using both pharmacokinetic and pharmacodynamic genetic markers in pharmacogenetic EPS risk assessment.

**Disclosure:**

No significant relationships.

